# Outcomes of early delivery in pregnant patients with acute respiratory distress syndrome

**DOI:** 10.1186/cc10719

**Published:** 2012-03-20

**Authors:** CY Hung, HC Hu, CH Chang, CC Huang, KC Kao

**Affiliations:** 1 Chang Gung Memorial Hospital, Taoyuan, Taiwan

## Introduction

Critical illnesses in pregnancy account for 0.11 to 0.89% of deliveries resulting in ICU admissions. The high rate of perinatal asphyxia in infants and high mortality rate in gravid patients supported a strategy of early delivery during the third trimester. The mortality rate of acute respiratory distress syndrome (ARDS) is high and varied from 15 to 72% among the studies. The present study reports the outcomes of early delivery within 48 hours after ICU admission in pregnant patients with ARDS.

## Methods

A total of 23 pregnant patients with gestational age more than 20 weeks admitted to the ICU was recorded from January 2009 to November 2012. Emergent delivery was performed within 48 hours after ICU admission. The collected data included etiologies of ICU admission, patients' characteristics and ventilator setting, infant and maternal clinical outcomes.

## Results

The gestational age more than 25 weeks was in 21 patients and between 20 and 25 weeks was in two patients. The mean age of these patients were 31 ± 5.7. The leading causes of ICU admission were obstetric emergency (26%), cardiovascular disease (26%) and infectious disease (26%) in these 23 patients. A total of 19 patients were respiratory failure and ARDS was diagnosed in nine of 19 patients. Of these nine ARDS patients, tidal volume (mean: 385 ± 31 ml), PaO_2_/FiO_2 _ratio (mean: 116 ± 47), positive end-expiratory pressure (PEEP) (mean: 13 ± 1.4 mmHg), peak airway pressure (mean: 34 ± 9.1 mmHg) and FiO_2 _(mean: 93 ± 7%). The intra-uterine fetal death ratio was 33% (3/9) and the Apgar score of the other six living births (6/9) was 7.8 ± 0.7. The hospital mortality rate of these ARDS patients was only 11% (1/9).

## Conclusion

For pregnant ARDS patients, intensivists had a challenge for fetal and maternal life-threatening distress. In our study, early delivery combined with a lung-protective ventilation strategy may provide significantly better fetal and maternal outcomes.
